# Progressive Tumefactive Demyelination as the Only Result of Extensive Diagnostic Work-Up: A Case Report

**DOI:** 10.3389/fneur.2021.701663

**Published:** 2021-07-08

**Authors:** Pavel Štourač, Jan Kolčava, Miloš Keřkovský, Tereza Kopřivová, Leoš Křen, Josef Bednařík

**Affiliations:** ^1^Faculty of Medicine, Masaryk University Brno, Brno, Czechia; ^2^Department of Neurology, University Hospital Brno, Brno, Czechia; ^3^Department of Radiology and Nuclear Medicine, University Hospital Brno, Brno, Czechia; ^4^Department of Pathology, University Hospital Brno, Brno, Czechia

**Keywords:** demyelinating diseases, multiple sclerosis, neuropathology, neuroradiology, case report

## Abstract

Tumefactive demyelinating lesions belong to the rare variants of multiple sclerosis, posing a diagnostic challenge since it is difficult to distinguish them from a neoplasm or other brain lesions and they require a careful differential diagnosis. This contribution presents the case report of a young female with progressive tumefactive demyelinating brain and spinal cord lesions. An extensive diagnostic process including two brain biopsies and an autopsy did not reveal any explanatory diagnosis other than multiple sclerosis. The patient was treated by various disease-modifying treatments without significant effect and died from ascendent infection via ventriculoperitoneal shunt resulting in *Staphylococcus aureus* meningitis.

## Introduction

Tumefactive demyelination belongs to the rare variants of multiple sclerosis (MS), posing a diagnostic challenge and therapeutic enigma as they can mimic other pathologies such as brain neoplasm, abscess, vasculitis, or granulomatous disease.

Atypical features of MS plaques on MRI include size >2 cm, mass effect, edema, and/or the presence of ringlike or open-ring enhancement. Lesions with these characteristics are often described as tumefactive demyelinating lesions (TDLs) (1).

The prevalence of TDLs is estimated to be one to three per 1,000 cases of MS (2), although Sánchez et al. report the prevalence of 21 per 1,000 cases of MS (3). Neuroimaging is necessary to confirm the diagnosis, and a biopsy may be warranted if imaging is not precise (4). The clinical presentation of patients with TDLs is variable and could be atypical for the demyelinating disease. The mass effect is usually the cause of symptoms due to the displacement of the surrounding tissue, is present in about half of TDL cases, and may lead to increased intracranial pressure and cerebral herniation (1). This contribution presents the case report of a young female with TDLs. Such a case report of a patient exhibiting similar TDLs has not been reported before.

## Case Report

**Figure 1:**
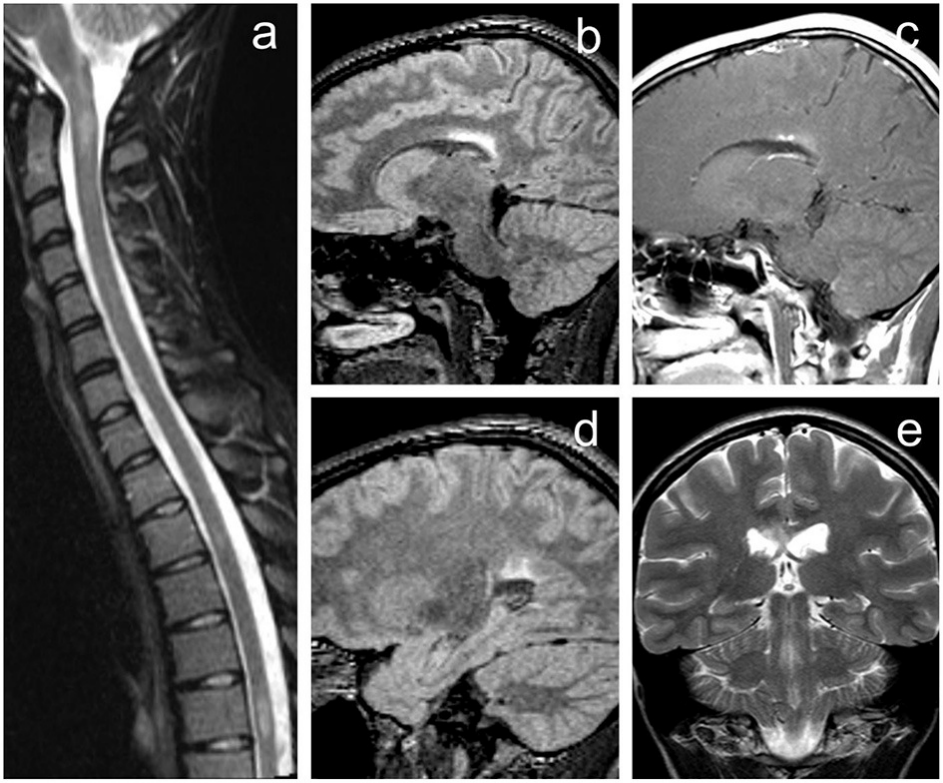
Initial MRI of the brain demonstrating rather typical finding of demyelinating disease fulfilling McDonald diagnostic criteria for multiple sclerosis. **(a)** Short-tau inversion recovery (STIR) image of the cervical and upper thoracic spine in sagittal plane revealing several hyperintense lesions (level C1/2, Th3/4, and Th6). **(b,d)** 3D fluid-attenuated inversion recovery (FLAIR) images of the brain in the sagittal plane. **(e)** T2-weighted image in the coronal plane. **(c)** Contrast-enhanced T1-weighted image with magnetization transfer in sagittal plane. Brain MRI revealed several periventricular white matter lesions including the largest FLAIR hyperintense plaque within the corpus callosum **(b,e)** with punctate enhancement **(c)**. Image **(d)** shows some of the other smaller lesions near the trigone of the left lateral ventricle.

A 23-year-old female was admitted to the neurological department of a major university hospital presenting with a mild central paraparesis of the lower extremities; an MRI indicated T2-hyperintense lesions in a periventricular, infratentorial, and intramedullary localization; both contrast-enhancing and non-enhancing lesions were found (Figure 1). Five oligoclonal bands (OCBs) appeared in the cerebrospinal fluid (CSF), but no OCB appeared in the serum; no signs of neuro-infection were found in the CSF. The patient's medical and family history was unremarkable, without any chronic disease, neoplasm, or autoimmune disease.

The MS diagnosis was determined according to the McDonald 2010 criteria (5). The patient was treated with high-dose steroids resulting in a slight reduction of complaints. Chronic treatment with interferon beta-1b commenced, and the patient was relapse-free for 4 years; no MRI progression appeared.

**Figure 2:**
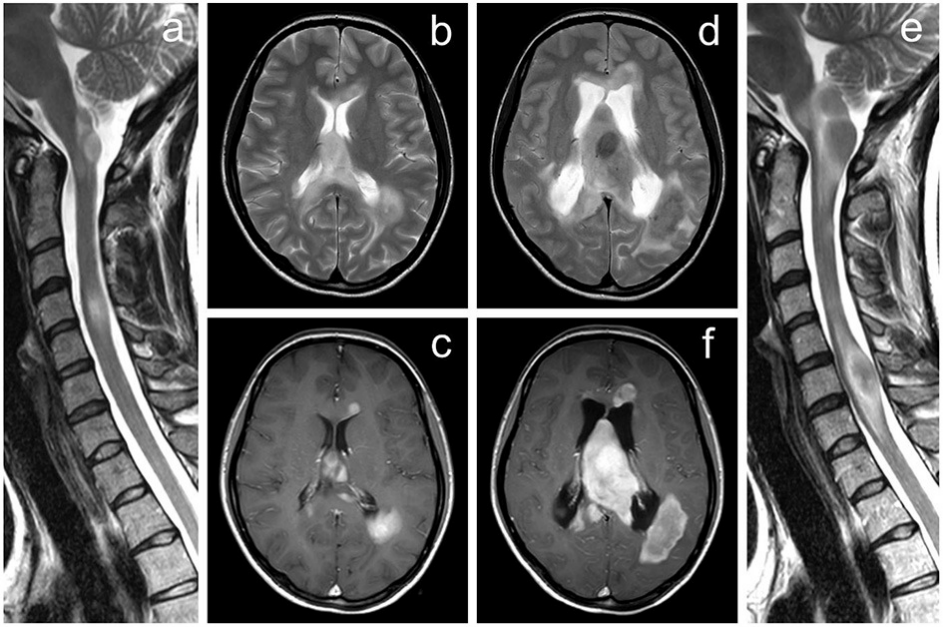
MRI of the brain and cervical spine documenting a significant progression of the tumefactive lesions. **(a–c)** Examination performed ~5 years after the initial diagnosis of multiple sclerosis, **(d–f)** follow-up examination after further 22 months. Several tumor-like enhancing lesions located around the lateral ventricles are visible on axial T2-weighted images **(b,d)** and contrast-enhanced axial T1-weighted images **(c,f)**. Extensive mass lesion located within the septum pellucidum finally caused the hydrocephalus by obstruction of the interventricular foramina; a significant dilatation of the lateral ventricles is seen on follow-up examination **(d,f)**. The progressive course of the disease is also demonstrated by T2-weighted images of the cervical spine in the sagittal plane **(a,e)**.

At the age of 27, the patient exhibited a mild central paraparesis of the lower extremities (treated with high-dose steroids), and the chronic treatment began with dimethyl-fumarate. An MRI of the brain and spinal cord showed multiple TDLs (Figure 2). An extensive diagnostic process was made, including positron emission tomography (PET) demonstrating high accumulation of 18F-fluoroethyl-L-thyrosine (FLT) within the lesions and MRI spectroscopy revealing elevation of choline peak and choline/creatine ratio. The histopathological findings from stereotactic brain biopsy of the lesion in the left occipital lobe confirmed demyelination; no neoplasm signs were found.

A broad range of tests was undertaken during follow-up (Table 1), all with negative results. The patient did not exhibit any other autoimmune disease or any other non-neurological manifestation over the whole follow-up period. She underwent a second CSF examination: no signs of infection or neoplasm were found, one OCB appeared in the CSF, and no OCB appeared in the serum.

**Table 1:** Laboratory tests undertaken during follow-up.

**Laboratory test categories**	**Detailed description**	**Result**
Autoimmune antibodies	Anti-aquaporin-4 antibodies, anti-myelin oligodendrocyte glycoprotein antibodies, anti-nuclear antibodies, antibodies against extractable nuclear antigens, anti-double- and single-stranded DNA, anti-neutrophil cytoplasmic antibodies, anticardiolipin antibodies, rheumatoid factor, anti-cyclic citrullinated peptide antibodies	Negative
Paraneoplastic antibodies (serum and CSF)	Anti-NMDAR, AMPA1, AMPA2, CASPR2, LGI1, GABAR B1, GABAR B2, anti-Hu, anti-Ri, anti-Yo, anti-CV2, anti-amphiphysin, anti-Ma1, anti-Ma2	Negative
Infectious diseases	John Cunningham virus (CSF), HIV, syphilis, toxoplasmosis, cryptococcal antigen (CSF), panfungal antigen	Negative
Metabolic disorders	Plasma amino acids analysis, urine amino acids analysis, plasma acylcarnitine analysis, urine sulfatides, plasma chitotriosidase, urine organic acids analysis, urinary acylglycines, serum very long-chain fatty acids analysis, serum carnitine quantification, serum purines and pyrimidines, serum homocysteine quantification, plasma creatine kinase, aspartate aminotransferase, alanine aminotransferase, lactate dehydrogenase, CSF lactate	Normal levels

*DNA, deoxyribonucleic acid; CSF, cerebrospinal fluid; NMDAR, N-methyl-D-aspartate receptor; AMPA, α-amino-3-hydroxy-5-methyl-4-isoxazolepropionic acid; CASPR2, contactin associated protein 2; LGI, leucine-rich glioma-inactivated; GABAR, gamma-aminobutyric acid receptor*.

At the age of 28, the patient exhibited left-sided negative sensitive symptoms and was treated with high-dose steroids. An MRI of the brain and cervical spinal cord showed significant progression of TDLs, and the treatment with natalizumab commenced.

**Figure 3:**
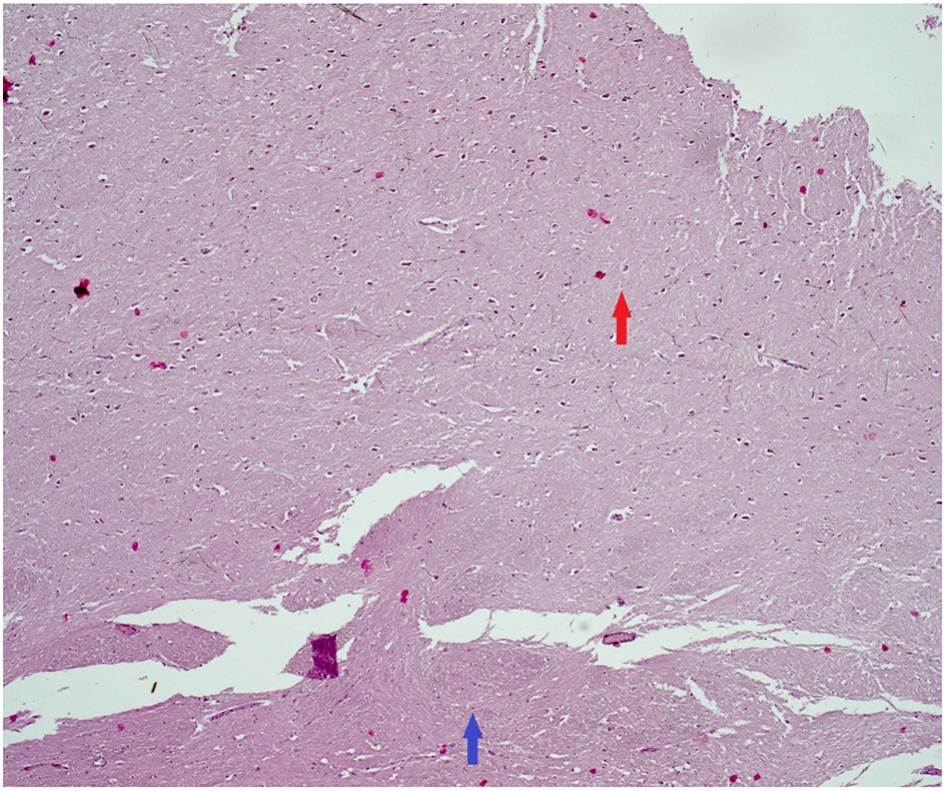
Histopathological findings (tumefactive lesion in the left occipital lobe). Hematoxylin–eosin. Red arrow: plaque with loosing of neuropil and reactive gliosis. Blue arrow: normal myelinated CNS tissue (50× magnification). CNS, central nervous system.

**Figure 4:**
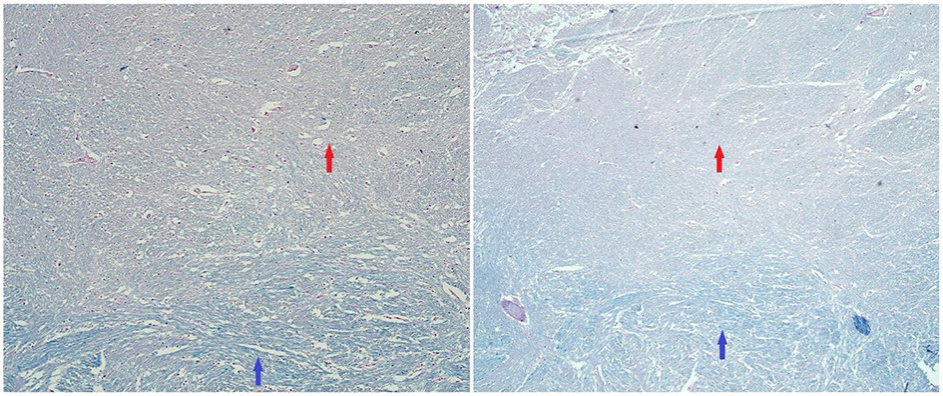
Histopathological findings (tumefactive lesion in the left occipital lobe). Luxol fast blue/periodic acid–Schiff (PAS) staining. Red arrows: area of demyelination in plaque (pinkish color of PAS). Blue arrows: areas of normal myelination (bluish staining of myelin by luxol fast blue) (100× magnification/50× magnification).

At the age of 29, cognitive and gait problems together with a headache and papilledema occurred, and a diagnosis of obstructive hydrocephalus (Figure 2) was established. Thus, a ventriculoperitoneal shunt was inserted. Brain MRI revealed further progression of TDLs, and the patient underwent another brain lesion biopsy. Histopathological findings revealed demyelination (Figures 3, 4).

At the age of 30, left-sided hemiparesis together with further progression of TDLs appeared. The patient was treated with high-dose steroids and an immunomodulatory dose of intravenous immunoglobulins resulting in a slight reduction of complaints to mild central paraparesis of the lower extremities and mild left-sided hemiparesis. From a chronic treatment point of view, hematopoietic stem-cell transplantation was considered. However, the patient died from circumscribed peritonitis complicated by ascendent infection via ventriculoperitoneal shunt resulting in *Staphylococcus aureus* meningitis. The autopsy confirmed the central nervous system demyelination without any evidence of neoplasm or other chronic disease.

## Discussion

This contribution presents the case report of a young female with TDLs. An extensive diagnostic work-up, including two biopsies and an autopsy, did not reveal any other explanatory diagnosis other than tumefactive MS. The report is unique due to subacute progressive TDLs of the brain and spinal cord, non-responsiveness to the high-efficacy drugs, and high diagnostic certainty due to multiple brain biopsies and an autopsy. Such a case report of a patient exhibiting similar TDLs has not been reported before.

In distinguishing between TDLs and other pathologies on MRI, the features listed above can be helpful along with follow-up imaging since TDLs tend to resolve in response to steroid therapy (6). However, the differentiation between TDLs and brain tumors by MRI alone may be difficult. One of the pathologies to be considered as a differential diagnosis of TDLs is primary central nervous system lymphoma (PCNSL) as cases of either concurrence of MS and PCNSL or demyelinating lesions preceding the development of PCNSL have been reported (7).

The value of advanced MRI techniques such as diffusion, perfusion imaging, or magnetic resonance spectroscopy has been studied in distinguishing between TDLs and brain tumors; however, it appears that those techniques still cannot provide definite diagnosis, and further studies are required to determine their additional value (8).

PET may also play some role in distinguishing TDLs from brain tumors (9). FLT tracer, which has been used in the diagnostic work-up in our patient, is generally referred to as a marker of proliferation in brain tumors; however, it should not be considered entirely specific as its increased uptake had also been observed in demyelinating lesions (10).

In the presented case, we observed several imaging features considerably atypical for TDLs like mostly homogenous enhancement, spectroscopy, and FLT-PET findings. Also, considering the constant progression of the mass lesions during the therapy leading finally to the development of obstructive hydrocephalus, the coincidence of tumor infiltration was suspected, and the brain biopsy was required to provide a definite diagnosis.

There is little comment on the effect of disease-modifying therapy on the evolution of TDLs. Some evidence supports that fingolimod should be avoided in MS patients with TDLs due to possible exacerbation (4). Patients with TDLs may have a better prognosis compared to MS patients without such lesions, especially when there is a good recovery from a tumefactive lesion (11, 12); however, there is a dearth of information on untreated TDLs in the literature.

## Data Availability Statement

The original contributions presented in the study are included in the article/supplementary material, further inquiries can be directed to the corresponding author/s.

## Ethics Statement

Ethical approval was not provided for this study on human participants because The manuscript presents a case report study. Written informed consent was not provided because According to the local ethics committees, the written informed consent is not necessary for a post-mortem case report study. Written informed consent was not obtained from the individual(s) for the publication of any potentially identifiable images or data included in this article.

## Author Contributions

All authors listed have made a substantial, direct and intellectual contribution to the work, and approved it for publication.

## Conflict of Interest

The authors declare that the research was conducted in the absence of any commercial or financial relationships that could be construed as a potential conflict of interest.
